# Exploring the health benefits of outdoor exercise for cancer survivors: a systematic review of more than 700 individuals

**DOI:** 10.1186/s13643-025-02834-y

**Published:** 2025-05-07

**Authors:** Sonia Ortega-Gómez, Luca Di Bartolo, Joanna Velissari, Beatriz Gomes, Susanna Pusa, Joshua Thaller, Sofia Papakonstantinou, Musa Kirkar, Ennio Iannitto, Nádia Moura, Carmen Nogueira, Jesús Gustavo Ponce-González, Rebecca Baxter, Paula Tavares, Apostolos Vantarakis, Antonino Bianco, Ana Carbonell-Baeza, David Jiménez-Pavón, the OACCUs consortium

**Affiliations:** 1 MOVE-IT Research Group, Department of Physical Education, Faculty of Education Sciences, Instituto de Investigación e Innovación Biomédica de Cádiz (INiBICA), Universidad de Cádiz, 11519 Puerto Real, Cádiz Spain; 2 Sport and Exercise Sciences Research Unit, Department of Psychology, Educational Sciences and Human Movement, University of Palermo, Palermo, Italy; 3 Department of Public Health, Medical School, University of Patras, Patras, Greece; 4 Centre for Innovative Biomedicine and Biotechnology (CIBB), University of Coimbra, Coimbra, Portugal; 5 Department of Nursing, Umeå University, Umeå, Sweden; 6 Department of Health Consulting, Research and Science, Outdoor Against Cancer, Munich, Germany; 7 Creative Thinking Development — CRE.THI.DEV, Rafina, Greece; 8 CEIPES ETS, Palermo, Italy; 9 Lega Italiana per la lotta Contro i Tumori (LILT Palermo), Palermo, Italy; 10 Portuguese Cancer League, Centre Branch, Coimbra, Portugal; 11 Innovation & Development Unit, Unidade Local de Saúde de Coimbra, Coimbra, Portugal; 12 Exphy Research Group, Department of Physical Education, Instituto de Investigación e Innovación Biomédica de Cádiz (INiBICA), Universidad de Cádiz, Cádiz, Spain; 13 CIBER of Frailty and Healthy Aging (CIBERFES), Madrid, Spain

**Keywords:** Exercise therapy, Quality of life, Survivorship, Mental health

## Abstract

**Background:**

Cancer’s rising incidence and growing survivor population underscore the need for strategies to enhance health and quality of life. Outdoor physical activity (PA) settings may provide unique benefits, yet evidence in this context is scarce. This systematic review aims to evaluate the impact of outdoor PA and exercise interventions on the health and well-being of cancer survivors.

**Methods:**

A systematic search was conducted in PubMed, Web of Science, and PsycINFO databases from their inception until April 23, 2024. Studies included randomized controlled trials (RCTs) and non-RCTs involving outdoor PA or exercise interventions for cancer survivors. The search strategy adhered to PRISMA guidelines, and the quality of studies was assessed using the PEDro scale.

**Results:**

Twelve studies involving 712 cancer survivors were included, comprising 7 RCTs and 5 non-RCTs. Four studies compared outdoor exercise to indoor exercise instead of a usual care control group, and one used a crossover design. The interventions varied in frequency, intensity, time, and type, with Nordic walking and walking being the most common. Key findings indicated significant improvements in mental health, PA levels, muscular fitness, body composition, and exercise motivation. However, the impact on vital signs and sleep quality was inconclusive due to the limited number of studies and variability in interventions.

**Conclusions:**

Outdoor PA, including structured exercise interventions, substantially benefits cancer survivors, particularly in enhancing mental health and physical fitness. Despite the promising findings, further research is needed to explore long-term effects, the benefits for different cancer types and age groups, and the underlying mechanisms of these interventions. Health practitioners should consider incorporating outdoor activities into cancer rehabilitation programs.

**Systematic review registration:**

PROSPERO CRD42024545392.

**Supplementary Information:**

The online version contains supplementary material available at 10.1186/s13643-025-02834-y.

## Keypoints

• Outdoor exercise improves both physical and mental health in cancer survivors. There is a limited evidence for outdoor exercise effects on sleep quality and vital signs.

• Interventions showed benefits, but progression and load variations need further study.

• Nordic walking improves upper body strength, mood, and pain self-efficacy. Adventure and water activities are the most studied after walking activities.

**Supplementary Information:**

The online version contains supplementary material available at 10.1186/s13643-025-02834-y.

## Introduction

Cancer represents a significant global health challenge, being one of the leading causes of morbidity and mortality worldwide. In 2020, approximately 19.3 million new cancer cases and 10 million cancer deaths were reported, according to GLOBOCAN data [1]. The incidence of cancer has shown a steady increase, attributed in part to an aging population and modifiable risk factors such as smoking, unhealthy diets, and physical inactivity. Concurrently, the number of cancer survivors has significantly increased; in the United States alone, it is estimated that there are over 16.9 million survivors, with this number projected to rise to 22.2 million by 2030 [2]. This growing population underscores the urgent need to identify and establish effective non-pharmacological strategies to improve the health and quality of life of these individuals, addressing the physical, psychological, and social sequelae resulting from cancer and its treatment.

Among the non-pharmacological strategies to improve the health and quality of life of cancer survivors, lifestyle-related interventions stand out prominently [3]. In particular, physical activity (PA) and structured exercise are among the most potent tools for enhancing a wide range of physical and mental health aspects [4]. PA includes any bodily movement that increases energy expenditure, while exercise refers to structured, repetitive activity aimed at enhancing fitness and health [5]. In the present study, the term PA encompasses both nonstructured activities and structured exercise interventions.

Systematic reviews have consistently demonstrated the beneficial effects of PA and exercise on cancer survivors. For instance, a meta-analysis by Fong et al. [6] concluded that exercise significantly improves physical function, reduces fatigue, and enhances quality of life. Moreover, a review by Buffart et al. [7] reported that exercise interventions lead to significant improvements in cardiorespiratory fitness, muscle strength, and mental health outcomes, including reductions in anxiety and depression. These findings underscore the critical role of PA and structured exercise as a cornerstone in the supportive care of cancer survivors, promoting both physical rehabilitation and psychological well-being.

Building on the importance of PA for cancer survivors, recent research has highlighted the beneficial effects of outdoor activities and nature contact on health [8, 9]. Numerous studies have demonstrated that exposure to natural environments can significantly enhance physical and mental well-being [10]. For example, a research agenda by Frumkin et al. [11] found that nature exposure is associated with reduced stress levels, improved mood, and enhanced overall well-being. Another study by Twohig-Bennett and Jones [12] concluded that green space exposure is linked to decreased risks of chronic illnesses, including cardiovascular disease and type II diabetes. Specifically, PA and exercise in natural settings have been shown to improve various health outcomes. A study by Thompson Coon et al. [13] revealed that outdoor exercise leads to greater feelings of revitalization, increased energy, and positive engagement while also reducing tension, confusion, anger, and depression compared to indoor exercise. Despite these promising findings, there is a notable lack of literature focusing on the application of this approach—PA and exercise in natural settings specifically for cancer survivors. This gap highlights the need for further research to explore the potential benefits of integrating nature-based activities into the supportive care of this growing population.

Considering the insights previously discussed, it can be suggested that PA and exercise conducted outdoors, particularly in natural settings, could serve as a highly beneficial strategy for enhancing the physical and mental health of cancer survivors [13]. This approach leverages the dual benefits of PA and nature exposure, which have both independently shown positive effects on health outcomes [14, 15]. Unfortunately, there is no clear consensus in scientific literature regarding the specific benefits of outdoor PA, including structured exercise, for cancer survivors. The existing studies are limited and vary widely in their methodologies, making it challenging to draw definitive conclusions. Therefore, the aim of this review is to systematically examine and analyze the existing evidence on intervention studies involving outdoor PA or exercise among cancer survivors and to assess its impact on their physical and mental health. This review seeks to fill the current knowledge gap and provide a clearer understanding of the potential benefits of this intervention strategy.

## Methods

### Protocol and registration

The systematic review followed the Preferred Reporting Items for Systematic reviews and Meta-Analyses statement (PRISMA) guidelines [16] shown in Supplementary Table S1. Eligibility criteria and analytical methods were specified a priori and entered into the International Prospective Register of Systematic Reviews database (PROSPERO reference number: CRD42024545392).

### Literature search strategy

The systematic search was conducted in the PubMed, Web of Science, and PsycINFO electronic databases, from their inception until April 23, 2024. The search terms included a combination of keywords related to the following topics: cancer disease (cancer, tumor, tumour carcinoma, oncology, metastasis, leukemia, leukaemia), exercise (exercise, training, PA, sport, movement, surfing, rock climbing, Nordic walking, sailing, plogging), and outdoor environment (outdoor, outside, nature, mountain, beach, sea, green space, blue health, blue care, park, garden, blue space, green gym, street). The connectors “OR” and “AND” were used to combine the search terms. Specifically, we used tags for searching in title, abstract, and keywords for PubMed search. As an example for the term “cancer,” we introduced (cancer [Title/Abstract]). Search strategies were adapted to each database and can be found in Supplementary Table S2. The electronic search was enhanced by manually examining the reference lists of pertinent publications to uncover further literature.

### Study selection and data extraction

Two authors (D. J. P. and S. O. G.) independently performed the study selection, and disagreements were resolved by discussion with a third reviewer (A. C. B.). To assess the level of agreement between the two primary reviewers, Cohen’s kappa coefficient (*κ* = 0.95) was calculated, indicating almost perfect agreement in study selection. Studies meeting each of the following criteria, according to the PICOS framework (participants, interventions, comparisons, outcomes, study design) [17], were selected for the systematic review: (i) cancer survivors under treatment and overcome; (ii) outdoor PA, including structured exercise intervention, but if an additional intervention (e.g., nutritional, cognitive) was included, it had to be identical in terms of frequency, duration, and content in both the outdoor PA group and its comparator (either an indoor PA group or a usual care control group, CG); (iii) studies comparing the outdoor intervention group with usual care CG or indoor PA intervention exclusively; (iv) assessing at least one health-related outcome; and (v) randomized controlled trials (RCTs) and non-RCTs. Gray literature (e.g., abstracts, conference proceedings, and editorials), case studies, reviews, and non-English documents were excluded from the analysis. We also excluded studies that included individuals without a prior history of cancer or those diagnosed with other diseases (e.g., cardiovascular or respiratory conditions). Studies comparing two types of outdoor PA interventions, as well as those with designs lacking a comparison group, were also excluded. Similarly, we excluded studies that combined PA with additional interventions (e.g., cognitive training) unless the same intervention was applied identically to both the outdoor PA group and its comparator (e.g., indoor PA or CG). This approach minimized potential confounding effects, increasing the likelihood that observed differences in outcomes were primarily attributable to the PA or exercise setting rather than to other factors.

Studies initially selected by the systematic search were preliminarily screened by title and abstract. The full text of those studies meeting the inclusion criteria was checked to elucidate their eligibility. The authors were contacted when necessary to clarify any uncertainties. Finally, studies meeting each of the following criteria were included in the systematic review. We collected the following data from each study, when available: (i) author’s name and year of study publication, (ii) study design, (iii) sample characteristics (including the number of participants, sex, age, and type of cancer), (iv) PA or exercise intervention (including the type, intensity, frequency, session length, duration and supervision of intervention), (v) endpoints of health, and (vi) main study results.

### Quality assessment and publication BIAS

Study quality was evaluated with the Physiotherapy Evidence Database (PEDro) scale, a valid measure of the methodological quality of clinical trials [18, 19]. It is composed of 11 items comprising external validity (item 1), internal validity (items 2 to 9), and statistical information (items 10 to 11). Items were scored as 1 (yes) and 0 (no) depending on whether the criterion was met in the study. The total PEDro score is obtained by adding the scores of items 2 to 11 to obtain a total score from 0 (lower quality) to 10 (higher quality) [18]. The authors propose that ratings of 0 to 3 are categorized as “poor,” 4 to 5 as “fair,” 6 to 8 as “good,” and 9 to 10 as “excellent” [18]. Three authors (A. C. B., J. G. P. G., and S. O. G.) independently scored the studies, and disagreements were resolved by discussion with a fourth author (D. J. P.).

## Results

### Study selection

**Fig. 1:**
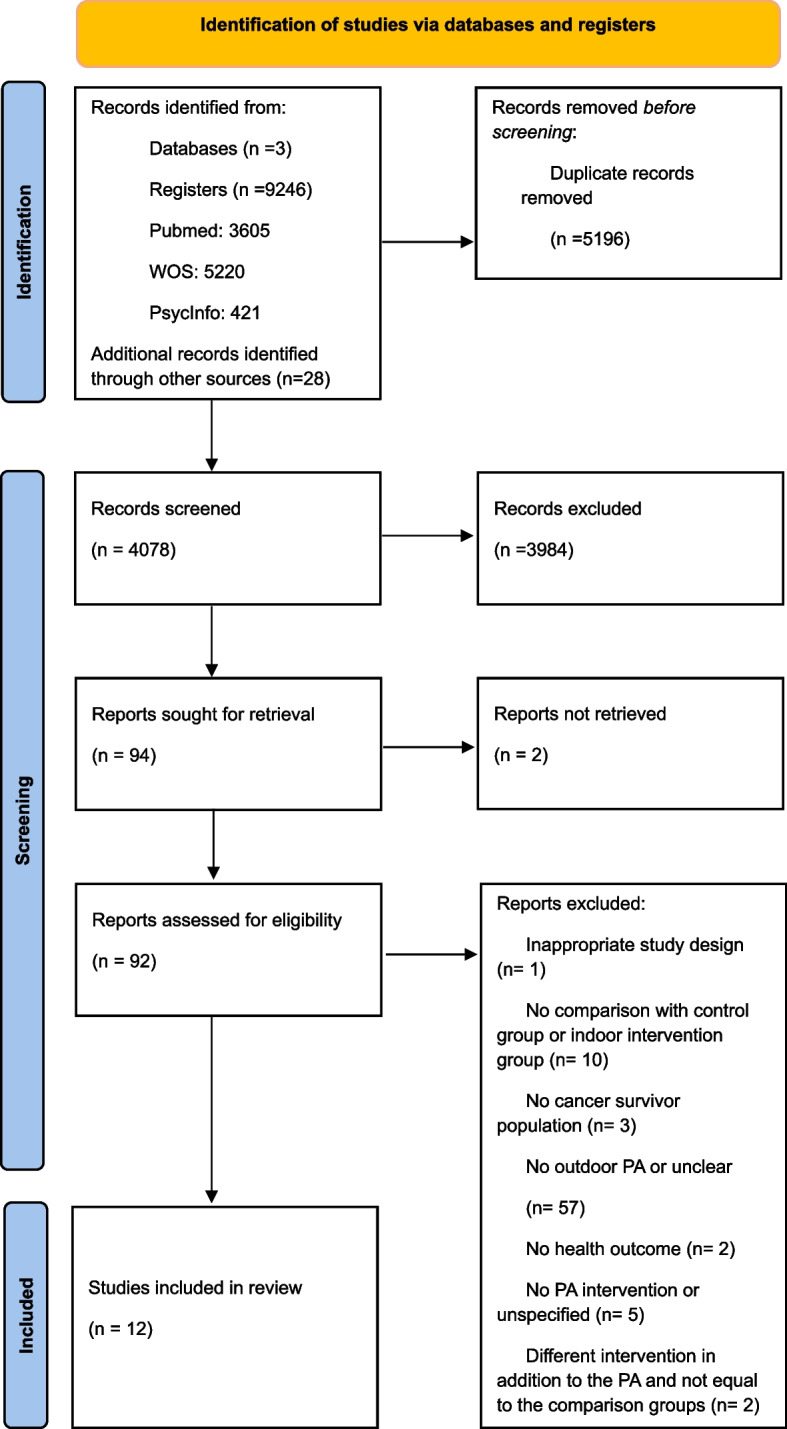
PRISMA flow chart of the selection process. Selection process for the systematic review. From 9246 records identified, 5196 duplicates were removed. After screening 4078 records, 3984 were excluded. Ninety-two reports were assessed for eligibility, with 80 excluded for various reasons. Twelve studies were included in the final review

A total of 9246 studies (PubMed: 3605; Web of Science: 5220; PsycINFO: 421) were identified through the electronic database search. Additional records were found from other sources (*n* = 28). Of these, 5196 duplicated studies were eliminated before screening. After checking the title and abstract, 94 full-text studies were selected for further review. Finally, after applying the criteria for inclusion and exclusion, 12 studies were included and evaluated in the present work [20–31]. The inter-rater reliability for the screening process was high (*κ* = 0.95), indicating almost perfect agreement between reviewers. See the flow diagram summarizing the selection process in Fig. 1.

### Quality assessment and publication BIAS

After the assessment of the publication quality by the PEDro scale, 25% (*n* = 3) of the studies were categorized as “poor” and 75% (*n* = 9) as “fair,” with no publication categorized as “good” or “excellent.” An overall overview depicting the studies meeting the quality criteria can be found in Supplementary material online, Supplementary Table S3.

### Study design

Of the studies included in this systematic review, seven were RCTs [20, 21, 24–28], and five were non-RCTs [22, 23, 29–31]. Four studies [20, 21, 29, 30] did not include a CG and instead compared outdoor exercise interventions with indoor exercise groups. Additionally, one study [21] employed a crossover design, where participants alternated between interventions.

As previously described, the methodological quality of the studies was assessed using the PEDro scale. While most studies were rated as fair, three non-RCTs [29–31] were classified as poor due to methodological limitations, such as lack of randomization. These differences in study design and quality should be considered when interpreting the findings.

### Participant’s characteristics

The characteristics of the 12 studies included are provided in Table 1. The total number of cancer survivors included was 712, and the number of participants per study ranged from 16 to 158. There was an overlap in two study samples [29, 30], so we only included one study from each case to calculate the overall number of cancer survivors. Six studies [20, 25, 26, 28–30] analyzed only women, while one [27] was focused only on men. The percentage of women ranged from 46 to 76% in those studies involving both sexes. In terms of age, this oscillated from 20 to 74 years old, and only three studies [21, 22, 31] were in young cancer survivors (< 40 years). Regarding the cancer types, 50% (*n* = 6) [20, 25, 26, 28–30] were based on breast cancer, and 21.4% (*n* = 3) included a wide variety of cancers [21, 22] or did not specify [31]. Specifically, Miller et al. [21] examined participants with leukemias (lymphoblastic and myeloid), central nervous system tumors, and other hematologic malignancies (Hodgkin’s lymphoma, post-transplant lymphoproliferative disease), pediatric tumors (Wilms tumor, Langerhans histiocytosis, aplastic anemia), and sarcomas. Gill et al. [22] included survivors of breast, non-Hodgkin’s and Hodgkin’s lymphoma, leukemia, brain tumors, thyroid cancer, and various other malignancies such as colon, ovarian, testicular, gastric, bone cancers, and sarcomas. In addition, multiple myeloma cancer [24], prostate cancer [27], and breast, bladder, testicular, and colon cancer [23] were represented within an 8.3% (*n* = 1) of studies for each category. On the status of cancer treatment, six studies reported that 100% of their sample was in the posttreatment phase [20, 21, 23, 24, 29, 30], four studies under treatment [25–28], one reported that 61% of their sample was in the posttreatment phase [22], and one indicated that 67% of their sample was under treatment [31].

**Table 1:** Main characteristics of the studies included in the systematic review

Author, year	Study design	Sample characteristics (*N*, sex, age)	PA/exercise intervention	Endpoints	Main results^a^
Carreño et al., 2023 [23]	Non-RCT	• *n* = 16 (14 women), 43–71 ys • Four rotational groups: EG- 1, EG- 2, EG- 3, CG • 88% women • Breast, bladder, testicle, and colon (N/A%) • 100% cancer post-treatment	*• Type*: EG- 1 (seafront walk with sea views), EG- 2 (snorkelling in the sea), EG- 3 (relax or swim in the sea), CG (sit and relax inside without views) • *Intensity*: Low • *Frequency*: Four sessions • *Session length*: 30 min • *Duration*: Four sessions per intervention (16 sessions in total) • *Supervised*: Yes	• *Well-being and mood state*: Overall Profile of Mood States (POMS) scores • *Arterial blood pressure and heart rate*: Sphygmomanometer • *Physical health parameters*: Heart rate and sleep quality by smartwatch Polar Vantage M Sleep quality parameters were as follows: total amount of sleep, sleep interruptions, sleep efficiency (%), deep sleep amount (%), and rapid eye movement (REM) sleep (%)	• ↑Well-being and better mood state in *EG- 1*, *EG- 2*, and *EG- 3* • No significant changes in heart rate or sleep quality
Czerwińska-Ledwig et al., 2022 [24]	RCT	• *EG*: *n* = 15 (8 women), 62.3 ± 8.5 ys • *CG*: *n* = 13 (5 women), 63.7 ± 3.7 ys • 46% women • 100% multiple myeloma • 100% cancer post-treatment	**EG** • *Type*: Nordic walking training • *Intensity*: 60–70% HRmax • *Frequency*: Three sessions/week • *Session length*: 60 min • *Duration*: Six weeks • *Supervised*: Yes **CG**: Any physical activity	*•Blood parameters*: Serum concentrations of vitamin 25(OH)D3, inorganic phosphorus, calcium, myoglobin, and lactate dehydrogenase	• ↓ Serum myoglobin concentration and ↑ in 25(OH)D3 and total calcium concentrations
Fields et al., 2016 [26]	RCT	• *EG*: *n* = 20 women,60 ± 8 ys • *CG*: *n* = 20 women, 66 ± 7 ys • 100% breast cancer • 100% cancer undertreatment	**EG** • *Type*: Nordic walking • *Intensity*: Low to vigorous • *Frequency*: One session/week • *Session length*: Sixty minutes (1–6 weeks), 4 × 30 min (7–12 weeks) • *Duration*: Twelve weeks • *Supervised*: 1–6 weeks (yes), 7–12 weeks (no) **CG:** Usual care	• *Primary outcomes*: Pain assessed by The Brief Pain Inventory, self-reported physical activity level using General Practice Physical Activity Questionnaire (GPPAQ), and adherence to exercise program by Macmillan Physical Activity Diary • *Secondary outcomes*: Depressive symptoms (Center for Epidemiological Studies Depression (CES-D)), self-efficacy to manage pain (Pain Self-Efficacy Questionnaire (PSEQ)), quality of life (Short Form Health Survey 36 (SF- 36))	*No statistically significant results (but clinically relevant)* • ↓Pain and depressive symptoms, and ↑self-efficacy pain and quality of life in *EG* after intervention and *CG* • ↑Vigorous PA by 39% in the *EG* and 15% in the *CG* during the intervention period • ↑Adherence to the intervention by the *EG* when supervised versus unsupervised • No changes in quality of life in the subscales of physical function and perception of general health in *CG*
Gill et al., 2016 [22]	Non-RCT	• *EG*: *n* = 50 (36 women), 31.1 ys • *CG*: *n* = 66 (52 women), 33.5 ys • 76% women • 35.2% breast • 10.3% Non-Hodgkin’s • 11.3% Hodgkin’s • 3.6% leukemia • 5.6% brain tumor • 7.3% Thyroid • 27% other (colon, ovarian, testicular, gastric, bone, carcinoma, sarcomas) • 61% cancer post-treatment	*Overall PA intervention* **EG** • *Type*: Outdoor venture program (whitewater kayaking, surfing, or rock climbing) • *Intensity*: N/A • *Frequency*: All days • *Session length*: 5–7 h per day • *Duration*: Seven days • *Supervised*: Yes **CG**: Usual care	• *Physical activity*: Seven-day physical activity recall • *Sedentary behaviors*: Two questions about number of hours spent watching television and seated in the previous 7 days • *Physical activity correlates*: The Sallis Self-Efficacy and Exercise Habits Survey, Environmental-Change Self-Efficacy Questionnaire, Perceived Barriers to Exercise Questionnaire, Preferred Activities Questionnaire, Enjoyment of PA Questionnaire, and Enjoyment of Inactive Recreation scale	• ↑PA (minutes/week) at both 1 week and 3-month follow-ups • ↓Hours of TV viewing and sitting time per week 1 week follow-up but not 3 months • ↓Excuses subscale (Perceived Barriers to Exercise) score 1-week follow-up but not 3 months • No changes in other PA correlates
Hanuszkiewicz et al., 2014 [29]	Non-RCT	• *EG- 1*: *n* = 20 women, 57 ± 8 ys • *EG- 2*: *n* = 20 women,60 ± 8 ys • *EG- 3*: *n* = 20 women,63 ± 8 ys • 100% breast cancer • 100% cancer post-treatment	• *Type*: EG- 1 (Nordic walking), EG- 2 (water resistance exercises), EG- 3 (general fitness exercise) • *Intensity*: 70–75% HRmax • *Frequency*: Two sessions/week • *Session length*: 45 min • *Duration*: 8 weeks • *Supervised*: N/A	*•Physical fitness*: Strength and velocity parameters of trunk flexor and extensor using an isokinetic test stand (Biodex System 3 Multi-Joint) at 60 and 120°/s	• ↑Trunk muscle function in *EG- 1* and *EG- 2* after intervention • No changes in trunk muscle function in *EG- 3* after intervention
Hanuszkiewicz et al., 2015 [30]	Non-RCT	• *EG- 1*: *n* = 20 women,57.3 ys • *EG- 2*: *n* = 20 women, 63.0 ys • *EG- 3*: *n* = 20 women,59.4 ys • 100% breast cancer • 100% cancer post-treatment	• *Type*: EG- 1 (Nordic walking), EG- 2 (water resistance exercises), and EG- 3 (general fitness exercise) • *Intensity*: 70–75% HRmax • *Frequency*: Two sessions/week • *Session length*: 45 min • *Duration*: 8 weeks •* Supervised*: N/A	*•Sagittal spinal curvatures*: ALPHA, BETA, GAMMA, TKA, LLA, and TIA using photogrammetry-based body posture tests with the use of Computer Body Posture Diagnosis device	• ↓The beta and alpha angles; ↑ in the thoracic kyphotic and lumbar lordotic angles in *EG- 1*. Balanced postural changes only in this group • ↓The beta angle and ↑ alpha, thoracic kyphotic and trunk inclination angles in *EG- 2* • No changes in* EG- 3*
Hanuszkiewicz et al., 2021 [20]	RCT	• *EG- 1*: *n* = 19 women, 51 ± 7 ys • *EG- 2*: *n* = 21 women,53 ± 8 ys • 100% breast cancer • 100% cancer post-treatment	• *Type*: EG- 1 (Nordic walking) and EG- 2 (general gymnastics) • *Intensity*: 65–70% HRmax • *Frequency*: Two sessions/week • *Session length*: 45 min • *Duration*: 8 weeks • *Supervised*: Yes	• *Sagittal spinal curvatures*: ALPHA, BETA, GAMMA, and TIA from Moiré-based imaging system • *Physical fitness*: Isokinetic trunk muscle endurance (total work and average power) using the Biodex multi-joint 3 isokinetic dynamometer	• ↓Sagittal spinal curvatures and ↑isokinetic trunk muscle endurance in *EG- 1* after intervention
Malicka et al., 2011 [25]	RCT	• *EG*: *n* = 23 women, 63.6 ± 6.8 ys • *CG*: *n* = 15 women, 63.8 ± 9.2 ys • 100% lymphoedema in breast cancer • 100% cancer undertreatment	**EG** • *Type*: Nordic walking • *Intensity*: 85% HRmax • *Frequency*: Two sessions/week • *Session length*: 60 min • *Duration*: 8 weeks • *Supervised*: Yes **CG**: No rehabiliation	• *Physical fitness*: Bilateral upper extremity strength using Biodex multi-joint 3 isokinetic dynamometer • *Volume of lymphoedema*: Circumferences and transformed using Limb Volumes Professional version 5.0	• ↑The function of the upper extremity muscles on the side treated • Nordic walking neither leads to the development of lymphoedema in women following breast cancer treatment nor does it intensify it
Miller et al., 2021 [21]	Randomized cross-over group pilot trial	• *EG- 1*: *n* = 9 (four women), 20.6 ys • *EG- 2*: *n* = 10 (six women), 19.9 ys • 53% women • 10.5% lymphoblastic leukemia • 10.5% myeloid leukemia • 10.5% central nervous system • 31.6% other: hematologic (Hodgkin’s lymphoma, posttransplant lymphoproliferative disorder), pediatric tumors (Wilms tumor, Langerhans histiocytosis, aplastic anemia), and sarcomas • Survivors of childhood cancer (100% post-treatment)	◦ *Type*: EG- 1 (two indoor walking sessions and two outdoor walking sessions) and EG- 2 (two outdoor walking sessions and two indoor walking sessions) • *Intensity*: 3.90 METs outdoor sessions and 3.27 METs indoor sessions • *Frequency*: Two sessions/week • *Session length*: 30–50 min • *Duration*: Four sessions (1 week of one intervention and then another week of the other intervention separated by a month) • *Supervised*: Yes	• *Physical activity*: Accelerometry • *Perceived autonomy, competence, and relatedness*: Psychological Need Satisfaction in Exercise Scale • *Exercise motivation*: Behavior’s Regulation in Exercise qQuestionnaire- 2 (BREQ- 2) • *Fatigue*: The Fatigue Scale-Adolescent (FSA) • *Attendance*: Number of sessions of each participant	• ↑Intensity during outdoor exercise sessions compared to indoor exercise sessions • No differences in fatigue during both types of sessions • No differences in any variable 2 weeks after both types of sessions, except extrinsic motivation (↑after indoor sessions and ↓after outdoor sessions)
Rosenberg et al., 2014 [31]	Non-RCT	• *EG*: *n* = 87 (76 women), 31.1 ys • *CG*: *n* = 71 (57 women), 29.3 ys • 87% women • Type of cancer N/A • 40–67% receiving treatment at pretest or post-test	*Overall PA intervention* **EG** • *Type*: Outdoor venture program (kayaking, surfing, or rock climbing) • *Intensity*: N/A • *Frequency*: All days • *Session length*: N/A • *Duration*: Six days • *Supervised*: Yes **CG**: Usual care	• *Body image*: Body Image Scale • *Self-compassion*: Self-Compassion Scale-Short Form • *Psychosocial function*: Psychological Screening Inventory- 2 (PSI- 2)	• Improved body image, self-compassion, self-esteem, and less discomfort, depression, alienation, fatigue/low energy, memory/concentration problems, and somatic anxiety symptoms • No differences in anger/aggression, anxious feelings, and verbally and socially outgoing scores
Uth et al., 2016 [27]	RCT	• *EG*: *n* = 29 men,67 ± 7 ys • *CG*: *n* = 28 men,67 ± 5 ys • 100% prostate cancer • 100% cancer undertreatment	**EG** • *Type*: Recreational football • *Intensity*: N/A • *Frequency*: 2–3 sessions/week • *Session length*: 45–60 min • *Duration*: 32 weeks • *Supervised*: Yes **CG**: Usual care	• *Body composition*: Total body lean mass, fat mass, fat mass percentage from DXA • *Body mass density*: Total hip, femoral shaft, femoral neck and lumbar spine, and systemic bone turnover markers from DXA • *Physical function*: Balance (stair climbing, bilateral stance, tandem stance, and flamingo test) and lower-limb muscle strength (1 RM in knee extension, countermovement jump test, and sit-to-stand test)	• ↑Total hip, femoral shaft body mass density, and lower-limb muscle strength
Yang et al., 2011 [28]	RCT	• *EG*: *n* = 19 women, 51 ± 7 ys • *CG*: *n* = 21 women • 53 ± 8 ys • 100% breast cancer • 100% cancer undertreatment	**EG** • *Type*: Home-based walking program • *Intensity*: 60–80% HRmax • *Frequency*: 2–3 sessions/week • *Session length*: 40 min • *Duration*: 12 weeks • *Supervised*: No **CG**: Usual care	• *Symptoms of treatment*: Anderson Symptom Inventory-Taiwanese version (MDASI-T) • *Mood status*: POMS Short Form • *Physical activity*: Self-reported level using 7-Day Physical Activity Recall	• ↑Mood status and symptoms

*CG* control group, *CRF* cardiorespiratory fitness, *EG* exercise group, *HRmax* maximum heart rate, *ISA* integrated supportive activity with foam balls, *MET* metabolic equivalent of task, *N/A* not applied, *PA* physical activity, *RCT* randomized controlled trial, *ys* years, *1RM* one maximum repetition, *↑* significant increase, *↓* significant decrease

^a^Only significant results were considered

**Fig. 2:**
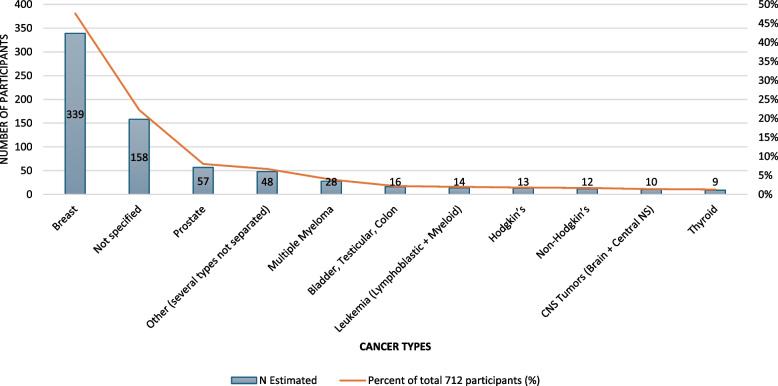
Distribution of cancer types among participants in the included studies. Breast cancer was the most represented, followed by cases where the specific cancer type was not reported. Other malignancies included prostate cancer, hematologic cancers, central nervous system tumors, thyroid cancer, and a category labeled “Other,” which comprised a variety of less frequently reported cancer types

The distribution of cancer types among the 712 participants included in this review is summarized in Fig. 2. Breast cancer was the most prevalent (47.6%), followed by cases where the specific cancer type was not reported (22.2%). Prostate cancer accounted for 8.0% of participants, while other malignancies, including hematologic cancers (e.g., Hodgkin’s and non-Hodgkin’s lymphoma, leukemia), central nervous system tumors, and thyroid cancer, were also represented in smaller proportions, 5.5%, 1.4%, and 1.3%, respectively. Additionally, 6.7% of participants were categorized under “Other,” which included a mix of less frequently reported cancer types such as colon, ovarian, testicular, gastric, bone cancers, and sarcomas. This heterogeneity highlights the diversity of populations studied in outdoor physical activity interventions for cancer survivors.

### Intervention characteristics

Based on the explicit definitions of PA and exercise and considering the reported characteristics of the included studies, it was determined that two studies [22, 31] were categorized as overall outdoor PA. These studies did not appear to involve structured interventions, as reflected in the descriptions provided. In contrast, the remaining studies were classified as exercise due to their more structured approach.

**Fig. 3:**
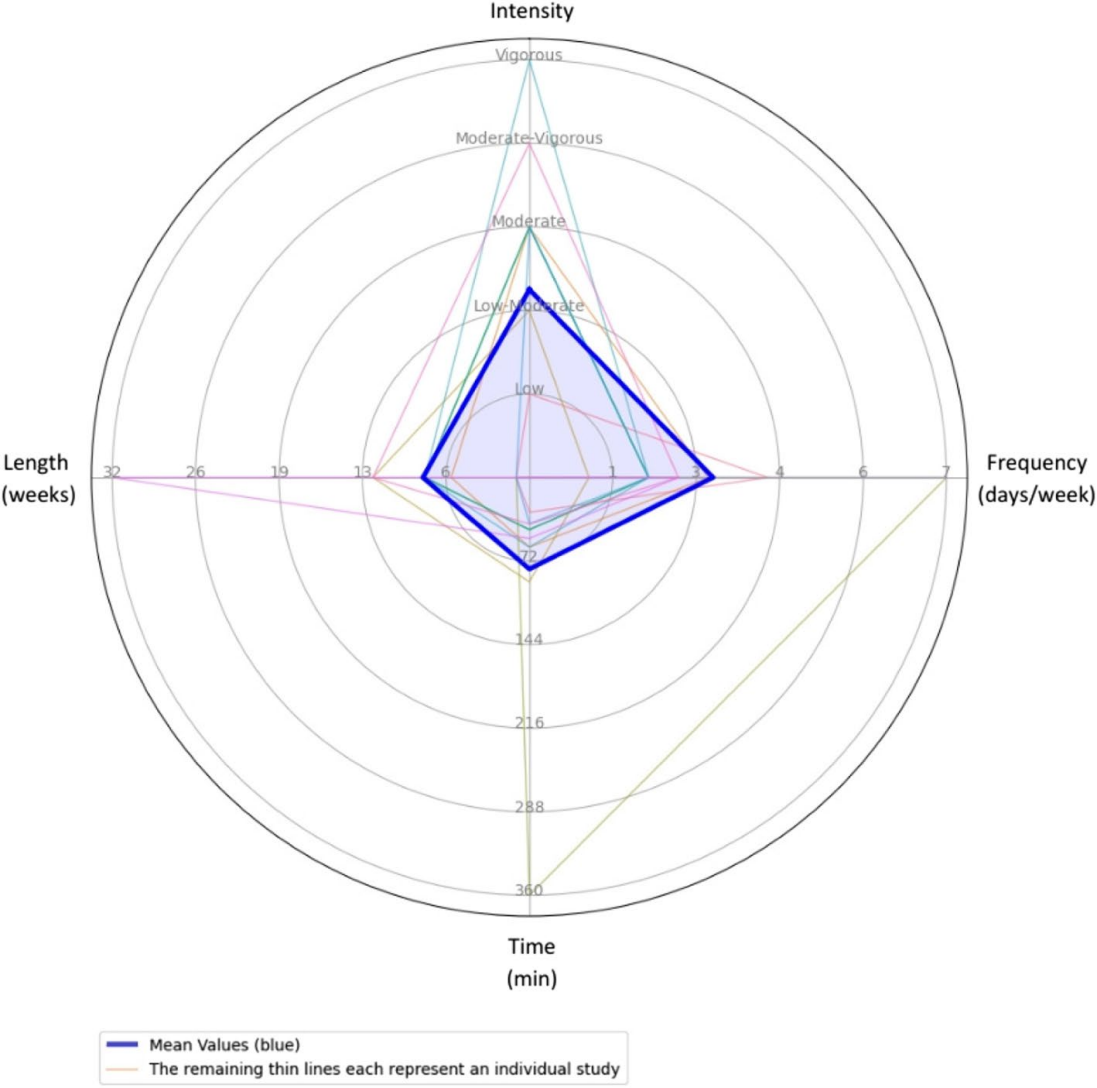
Radar chart with individual studies and mean values. Illustration of the intensity, frequency, time, and length of interventions across individual studies. Mean values are shown by the thick blue line, while thin lines represent individual studies

The *FITT* principle (frequency, intensity, time, and type) for exercise prescription was analyzed across all the included studies [32], and a summary of it is depicted in Fig. 3.

The *frequency* of sessions ranged from 1 to 3 sessions per week for 6 to 32 weeks of intervention in eight of the total studies. In contrast, the other four studies [21–23, 31] had 2 to 7 sessions in 1 week or did not specify the timing of the sessions [23].

The *intensity* reported by the studies was mostly in progression; one article [26] progressed from low to vigorous and four from moderate to vigorous [24, 28–30]. On the other hand, one article [23] indicated the low intensity of their intervention, two studies [20, 21] moderate intensity, and one article [25] vigorous intensity. Maximum heart rate (HRmax) or metabolic equivalent of tasks (METs) were the outcome variables. Intensity was not reported in two studies [27, 31].

The *times* of sessions ranged from 30 min to 2 h per day for eight of the total studies. In contrast, the other four studies [21–23, 31] had varied session times like 30–50 min per day, 5–7 h per day [22], or did not specify [31].

The *types* of outdoors PA and exercise interventions were classified into three categories: walking (with variants) (75%; 9 out of 12) [20, 21, 23–25, 28–30, 33], water and adventures activities (25%; 3 out of 12) [22, 23, 31], and regulatory sports (8.3%; 1 out of 12) [27].

More in detail, outdoor PA and exercise interventions consisted of walking [23, 28], Nordic walking [20, 24–26, 28–30], recreational football [27], physical activities in the sea such as snorkelling and swimming [23], and adventure programs related to aquatic activities and climbing [22, 31]. Conversely, the indoor exercise interventions carried out were water resistance exercise, general fitness exercise, and indoor walking.

The interventions were mostly supervised, except for two studies [29, 30] that did not report such information, one intervention [28] that was not supervised, and one intervention [21] that was half supervised.

### Intervention effectiveness by endpoints dimensions

**Fig. 4:**
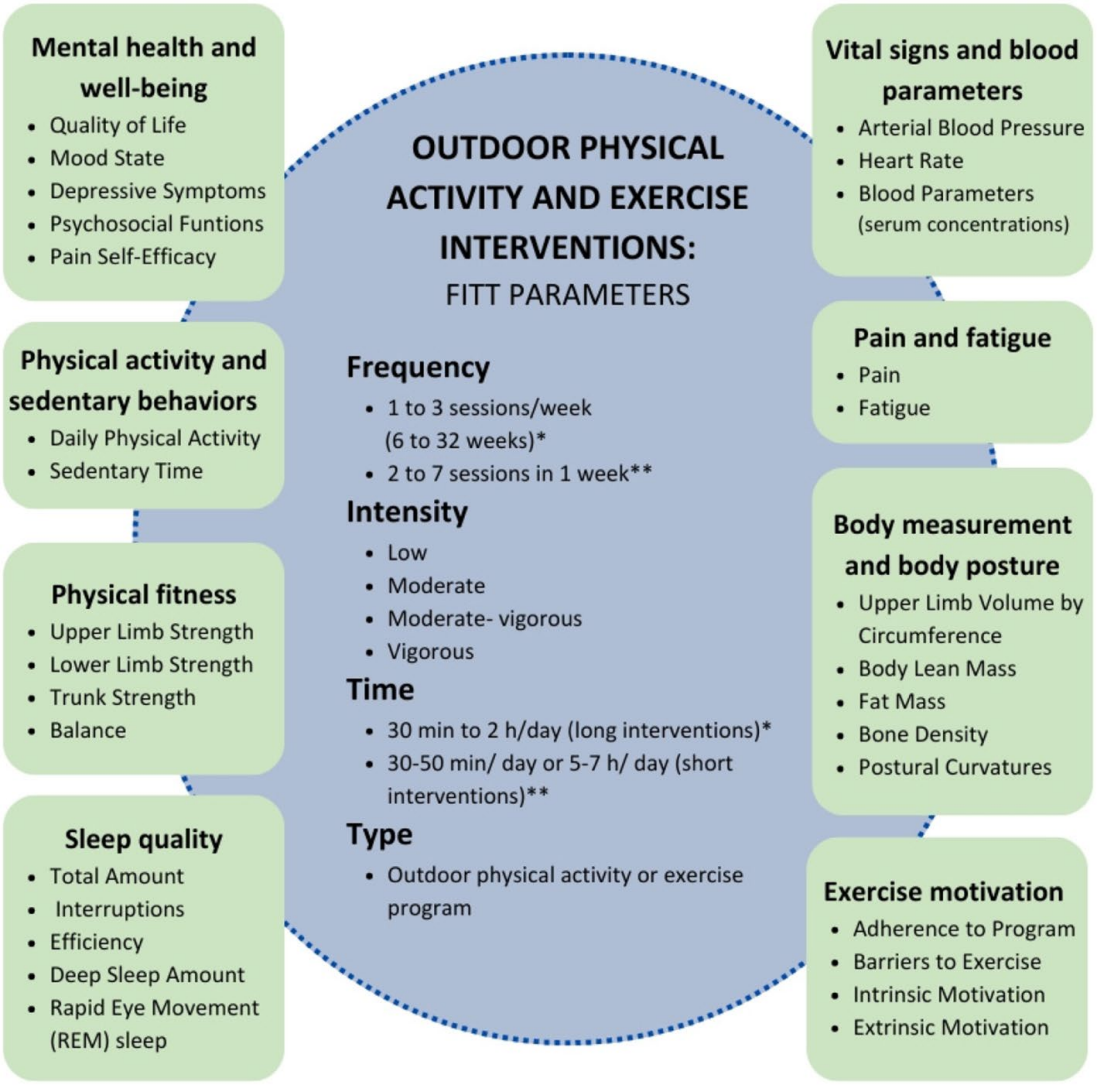
Classification of analyzed endpoint dimensions and FIIT parameters. Outdoor physical activity and exercise interventions categorized by analyzed endpoint dimensions and FIIT parameters. Dimensions include mental health and well-being, physical activity and sedentary behaviors, physical fitness, sleep quality, vital signs and blood parameters, pain and fatigue, body measurement and body posture, and exercise motivation. FIIT parameters cover frequency, intensity, time, and type of interventions. *Long interventions. **Short interventions

A total of eight dimensions were identified to classify all the endpoints analyzed in the different studies (Fig. 4).

#### Mental health and well-being

One study [26] assessed participants’ quality of life using the Short Form Health Survey 36 (SF- 36) showing that outdoor PA intervention of Nordic walking improved this quality of life to a clinically relevant higher magnitude than usual care. Moreover, the mood state obtained through the Profile of Mood States (POMS) was assessed in two studies [23, 28], which show that walking exercise interventions, walking with a sea view, and snorkelling or swimming in the sea improved the mood state as mental health indicator.

In addition, one study [26] assessed depressive symptoms using the Center for Epidemiological Studies Depression (CES-D) scale showing an improvement in symptoms clinically relevant after applying Nordic walking training, but without difference with the non-exercise group. Similarly, another study [31] assessed psychosocial function parameters using the Psychological Screening Inventory- 2 (PSI- 2), and their intervention of aquatic activities and climbing improved some psychosocial parameters compared to the no-walking group, including depressive symptoms and somatic symptoms of anxiety.

Finally, self-efficacy to manage pain, evaluated by Pain Self-Efficacy Questionnaire (PSEQ), was also studied [26] with a Nordic walking intervention, which showed clinically relevant improvement after the intervention although by the same magnitude as the non-exercise group. Body image using the Body Image Scale and self-compassion using the Self-Compassion Scale-Short Form were assessed in one study [31], and their aquatic and climbing intervention improved these parameters compared to the non-exercise group.

#### Physical activity and sedentary behaviors

Three studies assessed self-reported PA using the 7-day PA recall [22, 28] or the General Practice PA Questionnaire [26]. However, only one article [21] utilized objective assessment such as accelerometry. These three studies [21, 26, 28] whose intervention consisted of walking or Nordic walking showed no significant changes compared to the non-exercise group or the indoor walking group. Despite this, one study [26] describes a clinically relevant higher level of vigorous PA for the EG, with more than twice the amount compared to the CG. The study by Gill et al. [22] with an intervention based on a program of aquatic activities and climbing did improve the level of PA and even reduced sedentary time during the intervention and after 3 months of follow-up.

#### Physical fitness

Strength was assessed in four studies; one study [27] evaluated lower limb strength using several tests such as one maximum repetition in knee extension, countermovement jump test, and sit-to-stand test; another [25] assessed upper limb strength using the Biodex multi-joint 3 isokinetic dynamometer; and the last two [20, 29] assessed isokinetic trunk muscle endurance using the multi-joint 3 isokinetic dynamometer. All of them showed improvements after the intervention compared to the non-exercise group [25, 27] or a general fitness exercise indoor intervention [20, 29].

On the other hand, balance was assessed in the study by Uth et al. [27] using various tests such as stair climbing, bilateral and tandem stance, and the flamingo test. However, the recreational soccer intervention did not produce changes compared to the non-exercise group.

#### Sleep quality

The study by Carreño et al. [23] was the only one assessing sleep quality, by using the Polar Vantage M smartwatch, and the parameters were total amount of sleep, sleep interruptions, sleep efficiency, deep sleep amount, and rapid eye movement (REM) sleep. However, their exercise interventions at sea showed no difference compared to the non-exercise group.

#### Vital signs and blood parameters

The same study assessing sleep quality [23] analyzed vital signs such as arterial blood pressure and heart rate using sphygmomanometer and smartwatches (Polar Vantage M). Similarly, these parameters were also not better compared to the non-exercise group.

Blood parameters were also evaluated in another study with an intervention based on Nordic walking [24], in which serum concentrations of vitamin 25(OH)D3, myoglobin, and calcium showed better values.

#### Pain and fatigue

Body pain assessed by the Brief Pain Inventory-Short Form was studied [26] showing clinically relevant improvement after the Nordic walking intervention although with no difference against CG. Similarly, fatigue, assessed in one study [21] by the fatigue scale-adolescent, also showed no differences when comparing indoor and outdoor walking.

#### Body measurement and body posture

One study [25] assessed upper limb volume by circumference in breast cancer patients for the side effects of breast cancer and did not find a decrease in the volume of lymphoedema after Nordic walking intervention.

On the other hand, Uth et al. [27] analyzed body composition in depth by assessing total body lean mass, fat mass, percentage fat mass, and bone density at the total hip, femur, femoral neck and lumbar spine, and systemic markers of bone turnover. These measurements were determined by whole-body dual-energy X-ray absorptiometry (DXA) scan. Interestingly, there were only improvements in bone density after the recreational football intervention.

Finally, body posture was investigated in two studies [20, 30] from the same author, evaluating postural curvatures from the sagittal plane, as well as reflecting the angles ALPHA, BETA, GAMMA, TKA, LLA, and TIA of the spine. These assessments were performed using a Moiré-based imaging system, which analyzes spinal curvatures through optical measurements. Curvatures were less pronounced, and posture improved in the groups that performed Nordic walking and strength exercises in the water, but not in the group that performed general land fitness exercises.

#### Exercise motivation

The Fields’s study [26] examined adherence to their Nordic Walking program using the Macmillan PA Diary, and, although only from a clinical point of view, they found greater adherence in the supervised sessions in the first half of their intervention compared to the unsupervised sessions in the second half of their intervention.

In contrast, Miller et al. [21] only found improvements in extrinsic motivation, evaluated by Behaviors Regulation in Exercise Questionnaire- 2, after the indoor walking intervention compared to outdoor walking, despite also assessing perceived autonomy, competence, relatedness, and attendance. Gill et al. [22] also studied several parameters such as perceived barriers to exercise, preferred activities, enjoyment of PA, and enjoyment of inactive recreation, using validated questionnaires, including the Sallis Self-Efficacy and Exercise Habits Survey, the Perceived Barriers to Exercise Questionnaire, and the Enjoyment of PA and Inactive Recreation scales. However, the only significant finding was a reduction in the “excuses” subscale within the perceived barriers in the group that carried out the aquatic and climbing activities program.

**Fig. 5:**
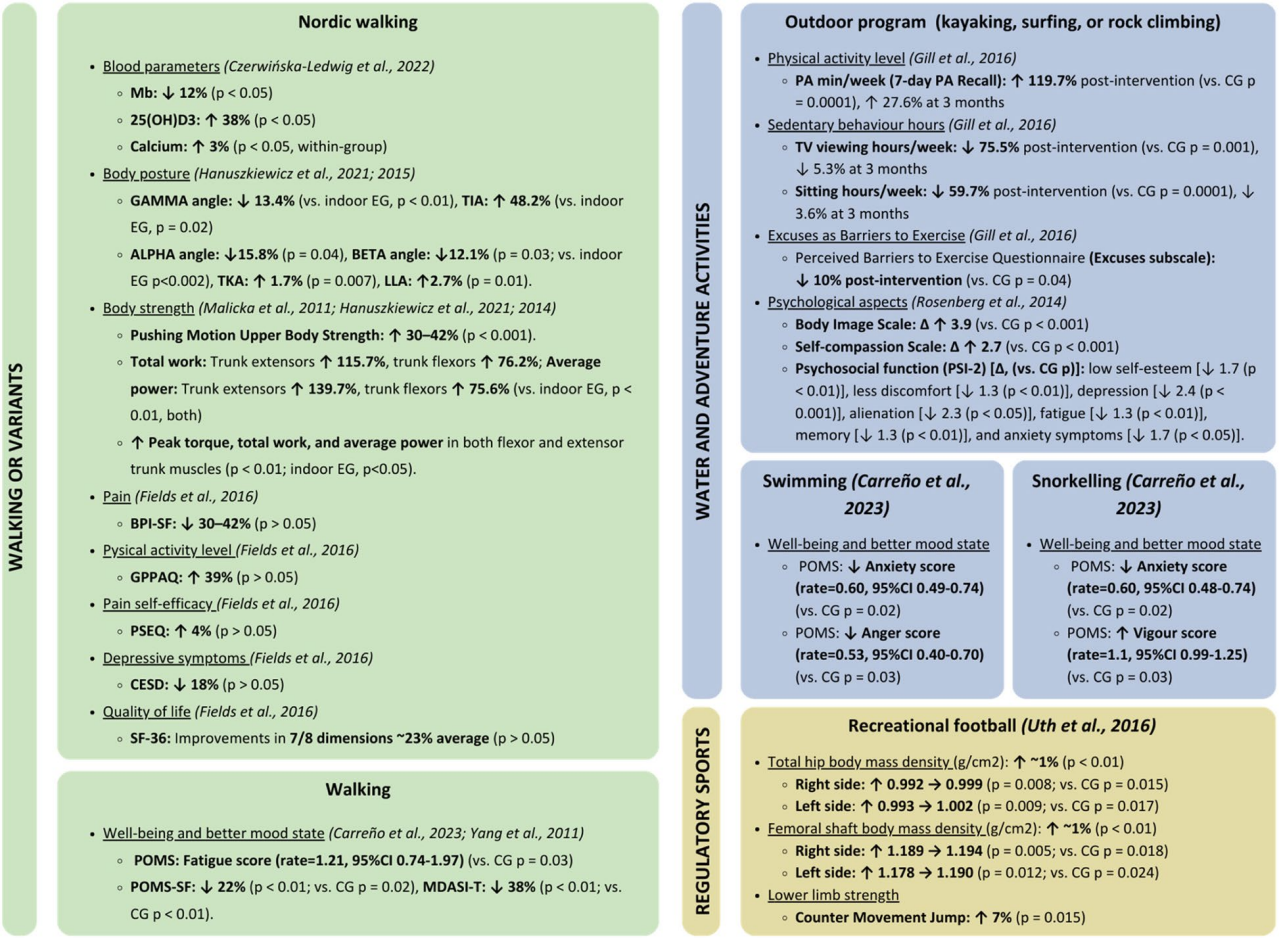
Benefits of outdoor physical activity by exercise type. Summary of the main benefits of outdoor physical activity interventions in cancer survivors, categorized by exercise type. Results are expressed as percentage changes (↑/↓) or absolute mean differences in pre-post intervention (Δ) with statistical significance (*p*-values). Abbreviations: 25(OH)D3, vitamin D metabolite; BPI-SF, Brief Pain Inventory-Short Form; CES-D, Center for Epidemiologic Studies Depression Scale; CG, control group; EG, exercise group; GPPAQ, General Practice Physical Activity Questionnaire; LLA, lumbar lordosis angle; Mb, myoglobin; MDASI-T, M.D. Anderson Symptom Inventory-Thyroid; PA, physical activity; POMS-SF, Profile of Mood States-Short Form; PSEQ, Pain Self-Efficacy Questionnaire; SF- 36, Short Form Health Survey; TKA, thoracic kyphosis angle

Additionally, a summary of the key findings classified by type of exercise interventions is provided in Fig. 5. Briefly, the walking category was one reporting a higher number of benefits, followed by the water and adventures category, although this one included a varied list of activities.

## Discussion

### Main findings

This systematic review aimed to evaluate the effects of outdoor PA and exercise interventions on the health and well-being of cancer survivors. The analysis of the included studies revealed several key findings concerning multiple health-related endpoints, which can be classified into eight dimensions. Briefly, the overall benefits of outdoor PA, including structured exercise, have been demonstrated for mental health and well-being, PA levels and sedentary behaviors, muscular fitness, body measurement and posture, and exercise motivation. Moreover, the average characteristics of the interventions are 3 days per week (*F*), low to moderate intensity (*I*), 79 min per session (*T*), and 8 weeks in duration. Finally, walking and Nordic walking were the most studied types (*T*) of outdoor exercise interventions, showing the strongest evidence of effectiveness. However, other activities, such as water-based and adventure activities, as well as regulated sports, also demonstrated beneficial effects. To our knowledge, this is the first systematic review to provide comprehensive evaluation of the impact of outdoor PA on multiple health dimensions in cancer survivors. This pioneering work consolidates evidence across various intervention types, offering novel insights into their effectiveness.

The improvement in mental health and well-being is one of the main findings. Specifically, outdoor physical activities such as Nordic walking consistently showed positive impacts on the mental health and quality of life of cancer survivors [26]. Several studies from this systematic review reported significant improvements in mood states [23, 28], reductions in depressive symptoms [26, 31], and enhanced self-efficacy for managing pain [26]. These positive benefits of outdoor PA have also been previously reported in other population groups and diseases [13]. However, this is the first comprehensive synthesis and analysis of all available evidence on the effectiveness of outdoor activities in cancer survivors.

In the case of the physical fitness dimension, only a few studies [20, 25, 27] considered this relevant dimension as an endpoint, and those particular studies demonstrated that outdoor PA interventions can lead to significant improvements in physical fitness parameters, particularly muscular strength. For instance, Nordic walking interventions [25, 26] were particularly effective in increasing upper body strength and vigorous PA levels. In this regard, physical fitness has been shown to be a very relevant health indicator in different population groups [34, 35] but also in cancer survivors due to its role in overall functionality [36, 37]. Thus, it is necessary to systematically include health-related physical fitness assessments in future intervention studies with outdoor activities to quantify their effectiveness on such a relevant dimension.

On the other hand, a limited impact of outdoor PA on vital signs and sleep quality has been found in this review. Specifically, the studied effects of outdoor PA on vital signs such as blood pressure and heart rate, as well as sleep quality, were less conclusive. A small number of studies [23, 24] assessed these parameters, and the results did not show significant differences compared to CGs. Nevertheless, previous evidence has reported an important influence of PA interventions on such vital signs [38] and sleep quality [39] in other population groups. For instance, the study performed by Baruki, Montebello, and Pazzianotto-Forti [40] found how outdoor PA improved vital signs in adults with hypertension. The limited number of studies conducted, along with the variability in intervention types considering these parameters in cancer survivors, may explain the lack of clear evidence on effectiveness.

In contrast, outdoor PA interventions showed promising results in improving body measurement and posture, particularly among breast cancer survivors [20, 25, 30]. Interventions involving Nordic walking and other physical activities led to improvements in postural alignment. These findings are partially supported by others who previously found Nordic walking improves body posture in older adults [41].

A further key contribution of this review is its analysis of the FITT characteristics of outdoor PA interventions, providing valuable insights for exercise prescription in cancer survivors. While previous studies have focused primarily on indoor or supervised clinical settings, this review offers a new perspective on how structured outdoor programs can be effectively implemented. From the perspective of exercise prescription and FITT characteristics analyzed in the interventions, the applied criteria showed that a standard design regarding frequency, intensity, time, and type of exercise was sufficient to obtain a significant list of physical and mental benefits. However, further research is needed to focus on the specific roles of progression and variation in the parameters of volume and intensity load, in line with recent suggestions [42], as well as better monitoring of physiological responses, as this could enhance the positive impact on the health of cancer survivors.

Despite the positive outcomes observed, there are notable gaps in the literature that need to be addressed in future research. Most of the studies from this review assessed short-term interventions. Future research should focus on the long-term effects of outdoor PA on health outcomes in cancer survivors. Additionally, there is very little evidence on young cancer survivors, highlighting the urgent need for research on this age group, as it is becoming a highly relevant problem at the international level. Moreover, the majority of the studies focused on breast cancer survivors; thus, more research is needed to understand the benefits of outdoor physical activities for survivors of other cancer types and diverse demographic groups.

A major challenge in synthesizing the findings of this systematic review was the considerable heterogeneity among the studies included. The heterogeneity in intervention characteristics (FITT principle), participant demographics, outcome measures, and methodological quality posed significant challenges in drawing definitive conclusions. Specifically, the broad range of cancer types and stages, combined with variations in intervention intensity and supervision, likely contributed to the inconsistencies in reported effects. Due to these discrepancies, a meta-analysis was deemed unfeasible, as statistically pooling such diverse data could have resulted in misleading conclusions. Instead, a narrative synthesis was conducted to summarize the observed trends across studies.

A notable limitation of this review is the variability in how the included studies controlled for potential confounding factors, such as cancer type, treatment status, and baseline physical fitness. While some studies accounted for variables like age, gender, weight, medication use, and baseline physical activity levels, others did not, which may have influenced the reported effects. Additionally, psychological factors such as anxiety, stress, and depression may interact with the effectiveness of outdoor PA interventions. As noted by Yang et al. [28], cancer survivors may gradually adapt to distressing symptoms, which could attenuate the observed benefits. To enhance the reliability of findings and allow for future meta-analytic approaches, research should strive for greater standardization in study designs, outcome assessments, and the control of key confounders.

Overall, the findings of the current review suggest practical implications that incorporating outdoor PA, including structured exercise interventions, into the supportive care of cancer survivors can offer substantial benefits. Health practitioners should consider recommending outdoor activities as a complementary strategy to enhance the physical and mental well-being of cancer survivors. Programs designed to encourage regular participation in outdoor activities could be integrated into cancer rehabilitation protocols, especially under the supervision of physical educators who would ensure the appropriate implementation and monitoring of the FITT principle.

Finally, the underlying mechanisms through which outdoor PA improves health outcomes in cancer survivors are not well understood. Consequently, studies exploring the specific physiological and psychological pathways involved would be beneficial. The added value of this review also lies in identifying critical research gaps. While outdoor PA interventions show promising results, there is a lack of long-term studies and standardized methodologies to compare outcomes across different cancer types and age groups. Future research should address these gaps to optimize outdoor PA interventions for diverse populations.

This review has several strengths, including a comprehensive search strategy, rigorous inclusion criteria, and a detailed analysis of intervention characteristics and outcomes. By compiling evidence on outdoor PA, this study provides a foundation for future clinical guidelines that integrate outdoor activities into cancer rehabilitation programs. However, there are also limitations to consider. One of these is the diversity of outdoor PA interventions and the variability in their implementation, making it challenging to draw definitive conclusions about their effectiveness. Moreover, the methodological quality of the included studies varied, with many studies categorized as “fair” or “poor” according to the PEDro scale, affecting the reliability of the findings.

## Conclusions

Outdoor PA, including structured exercise interventions, appear to be beneficial for both the physical and mental health of cancer survivors. Despite the limitations in the existing literature, the evidence supports the integration of outdoor exercise into cancer rehabilitation programs supervised by physical educator professionals as part of a community program (phase III) with holistic benefits for patients. Further, high-quality research is needed to explore the long-term effects and mechanisms of these interventions, as well as their applicability to a broader range of cancer types and populations, such as young cancer survivors and older adults with cancer among others.

## Supplementary Information

Supplementary Material 1: Supplementary Table S1. PRISMA guidelines checklist for the present systematic review. Supplementary Table S2. Search terms and search strategies to each database. Supplementary Table S3. Quality assessment with PEDro scale of included studies

## Abbreviations

CG: Control group
CES-D: Center for Epidemiologic Studies Depression Scale
DXA: Dual-energy X-ray absorptiometry
FIIT: Frequency, intensity, time, and type
HRmax: Maximum heart rate
MET: Metabolic equivalent of task
PA: Physical activity
PEDro: Physiotherapy Evidence Database scale
POMS: Profile of mood states
PSEQ: Pain Self-Efficacy Questionnaire
RCT: Randomized controlled trial
REM: Rapid eye movement
SF- 36: Short Form Health Survey 36
